# When and where to intervene: identifying key school segments to enhance adolescent physical activity

**DOI:** 10.3389/fspor.2026.1716537

**Published:** 2026-02-17

**Authors:** Marion Gasser, Andrea-Maria Nadenbousch, Fabienne Egger, Mario Kamer, Stefan Valkanover, Mirko Schmidt

**Affiliations:** 1 Institute of Sport Science, University of Bern, Bern, Switzerland; 2 Centre of Subject Didactics, Bern University of Teacher Education, Bern, Switzerland; 3 Institute for Primary Education, Bern University of Teacher Education, Bern, Switzerland; 4 Institute for Lower Secondary Education, Bern University of Teacher Education, Bern, Switzerland

**Keywords:** adolescents, leisure time, physical activity patterns, school segments, secondary school

## Abstract

**Background:**

Swiss adolescents fall short of the WHO's guideline of 60 min of moderate-to-vigorous physical activity (MVPA) per day. Developing targeted interventions or policies requires an understanding of adolescents’ daily activity patterns. Since adolescents spend much time at school, it is essential to consider not only leisure but also school segments when assessing physical activity (PA). Therefore, this study investigates how Swiss adolescents’ PA is distributed across different school time segments and examines to what extent they meet recommended activity levels.

**Methods:**

This cross-sectional study uses baseline data from the Active School project. The sample included 666 7th-grade students (mean age = 13.27 ± 0.55 years, 47.7% boys, 51.8% girls, 0.5% diverse) from 12 secondary schools. PA data, gathered over five schooldays using wrist-worn GENEActiv accelerometers, were segmented into physical education (PE), recess, classroom time, entire school time, and leisure time. Activity levels were categorized into inactivity (IN), light physical activity (LPA), and MVPA. Descriptive and inferential statistics (ANOVAs, *t*-tests) examined differences across segments and gender.

**Results:**

Within school time, MVPA varied significantly by segment (PE: 30.59%, recess: 18.80%, classroom: 5.69%, *p* < .001) and remained below recommendations for PE (50%) and recess (40%). IN dominated all segments, especially classroom time (75.83%). Overall, only half (49.5%) of Swiss adolescents met the school-based PA recommendation of at least 30 min of MVPA per day, while girls accumulated less MVPA and more IN than boys across all school segments (all *p*s < .001).

**Conclusion:**

Substantial opportunities for PA are lost across all school segments in the Swiss context, with girls consistently less active than boys. Based on these findings, segment-specific and gender-sensitive school PA policies are discussed, and a comprehensive school approach to PA promotion is recommended to support more effective and equitable PA promotion among adolescents.

**Trial Registration:**

German Clinical Trials Register (DRKS00033362). Date of registration: January 25, 2024. Retrospectively registered.

## 1 Introduction

The importance of physical activity (PA) for children and adolescents is widely recognized due to its positive effects on physical and mental health, cognitive function, and academic performance (1–3). Despite these well-documented benefits of regular PA, many children and adolescents remain insufficiently active (4, 5). The decline in PA is especially evident during the transition to adolescence, with longitudinal studies reporting annual decreases of approximately 3%–5% in moderate-to-vigorous physical activity (MVPA) (6, 7). Estimates for European countries indicate that at least 75% of adolescents fail to achieve the WHO's recommendation of 60 min of MVPA per day, but considerable variation exists among countries (8). Although Swiss adolescents demonstrate higher PA levels than European peers (9), every second adolescent still falls short of meeting the national PA guidelines (10, 11). This underlines the importance of addressing insufficient PA even in countries with comparatively favorable activity levels.

Understanding how adolescents accumulate PA across different times of the day is essential for identifying where and when promotion efforts should be targeted (12, 13). A typical weekday can be divided into unstructured and structured time periods (14). Unstructured time refers to more flexible parts of the day, such as leisure time, where adolescents have greater autonomy to be physically active. In contrast, structured time includes periods like classroom instruction or scheduled school activities that are externally regulated. International studies consistently show that adolescents accumulate more PA during leisure time than during school hours (14–16). These findings suggest that the structured school setting may displace opportunities for PA (17). However, a direct comparison with PA levels during leisure and school time has not yet been conducted in the Swiss context.

Given the generally low levels of PA during school time, schools are widely seen as a key setting for PA promotion, as they provide access to almost the entire adolescent population and engage them for a large part of the day (18). Traditionally, physical education (PE) has been considered the primary context for such promotion (19). In Switzerland, PE is a compulsory subject with three lessons per week (135 min), and, unlike in many other countries, teachers receive specific PE training as part of their teacher education (20, 21). Despite these favorable structural conditions, Swiss students spend the majority of their school day inactive (63%), with only around 5% of school time spent in MVPA (22). Accordingly, national recommendations advocate extending PA promotion beyond PE to other segments of the school day (23, 24). This aligns with international research pointing towards comprehensive school approaches (25–27). To this end, other school segments, such as recess (e.g., access to equipment) or classroom time (e.g., activity breaks or physical active learning), have also been suggested as important contributors to adolescents’ PA accumulation within the school environment (28).

With growing recognition of comprehensive school approaches as effective strategies to promote PA, segment-specific recommendations within the school day have gained increased attention in recent years (29). This is particularly relevant, as research indicates that schools implementing segment-specific PA guidelines report higher levels of school-based PA than those without such policies (30, 31). According to international guidelines, adolescents should engage in at least 30 min of MVPA during the whole school day, corresponding to half the daily recommendation (32). Furthermore, at least 50% of PE class time is suggested for MVPA (33), given that PE classes are the only compulsory and structured part of the school day explicitly dedicated to PA (34). For recess, 40% MVPA is recommended to support meaningful PA accumulation (35). Despite occupying the largest portion of the school day, classroom time currently has no official PA recommendations, likely because its primary focus is on academic learning. Nevertheless, classroom organization can offer opportunities for short movement breaks that may also enhance learning outcomes (36).

International research has increasingly investigated compliance with school-based PA recommendations among adolescents (29). Systematic reviews of PE lessons indicate that fewer than half of students meet the recommendations, with an average of 34–40% of lesson time spent in MVPA (37, 38). Similarly, compliance during recess is low, with MVPA levels ranging between 10–17% (16, 39), while inactivity (IN) and light physical activity (LPA) account for a substantial portion of this segment. Regarding classroom time, few studies exist, but available evidence suggests that adolescents spend 80–86% of this time inactive or engage in only about 5% MVPA (39). Taken together, these findings highlight that less than a quarter of adolescents meet the school-based recommendation of at least 30 min of MVPA per day (40).

In Switzerland, only a limited number of studies have examined compliance with school-based PA recommendations across specific school segments. Existing research has primarily focused on overall PA levels throughout the entire school time and on PE (22, 41–43). Findings indicate that Swiss students accumulate an average of only 21 min of MVPA during school hours, with just 14% meeting the school-based recommendation of at least 30 min (22). Even during PE, most students do not meet the PA recommendations (41), with an average of 32.8% of the lesson spent in MVPA (42). However, these studies were conducted among primary school children. Analysis in adolescents attending secondary schools is still lacking, and no studies to date have examined PA patterns during recess and classroom time, highlighting an important gap in the current evidence base. As a result, it remains unclear which school segments offer the greatest potential for targeted PA promotion and future intervention strategies. This is particularly relevant given that school-based interventions in Switzerland, such as the *KISS* study (44, 45) or the national program *School in Motion* (46), have implemented PA-promoting strategies across different school segments (e.g., PE, recess, and classroom time), yet without a detailed analysis of segment-specific needs or evidence of segment-specific effects.

Beyond the analysis of PA behavior within school segments, Swiss studies among primary school children have highlighted gender-based disparities, with girls tending to be less active than boys throughout the school day (22). Therefore, it is important to note that although segments such as PE, recess, and classroom time provide all students with the same structural conditions and environmental opportunities to be physically active, actual PA engagement can vary considerably between individuals (12, 16). International evidence also consistently demonstrates gender differences in PA levels, with boys typically engaging in more physical activity than girls across school and leisure time segments (14, 39). These differences are attributed to both biological and psychosocial changes during maturation, as well as the influence of socially constructed gender roles (47, 48). For instance, PE lessons often emphasize activities such as ball games and competitive formats, which tend to align more with boys’ interests and may discourage girls from active participation (49, 50). However, whether similar gender-based differences exist among Swiss adolescents within different school segments remains unclear, which would provide an important basis for refining future intervention strategies.

This study therefore pursues three main aims: (1) to compare PA behavior during leisure time and school hours to assess whether the school setting limits adolescents’ opportunities to engage in PA; (2) to analyze adolescents PA behavior (IN, LPA, and MVPA) across different school segments (PE, recess, classroom time) and evaluate compliance with school-based PA recommendations; (3) to examine gender differences in PA behavior across the time segments. Based on the existing literature, we hypothesized that PA percentage levels would be lower during school hours compared to leisure time, that most adolescents would not meet school-based PA recommendations, and that girls would be less active than boys across all segments.

## 2 Materials and methods

### 2.1 Study design and setting

This study adopts a cross-sectional design and is based on baseline data from the Swiss Active School project (German Clinical Trials Register, DRKS00033362), which evaluates a comprehensive school-based physical activity program. A study protocol has been published elsewhere (51). Baseline data were collected between 2023 and 2024 in 12 secondary schools in the canton of Bern, Switzerland. Schools were eligible for inclusion if they were public or semi-public secondary schools, including at least two classes per grade level, were not specialized (e.g., talent-, arts-, or boarding schools), and were not involved in major school-based PA promotion projects. The two measurement periods were scheduled during the summer months to ensure comparable PA assessments (52). Bern was chosen for its geographical diversity, which reflects the broader Swiss context; accordingly, both urban and rural schools were included to ensure a representative sample of Swiss adolescents. Parents of participating students provided written informed consent. Before the first data collection, all students were asked if they wished to participate and informed that they could withdraw at any time. All collected data were handled confidentially. Ethical approval was granted by the Ethics Commission of the Faculty of Human Sciences at the University of Bern (Ref: 2023-03-05).

### 2.2 Participants

**Figure 1:**
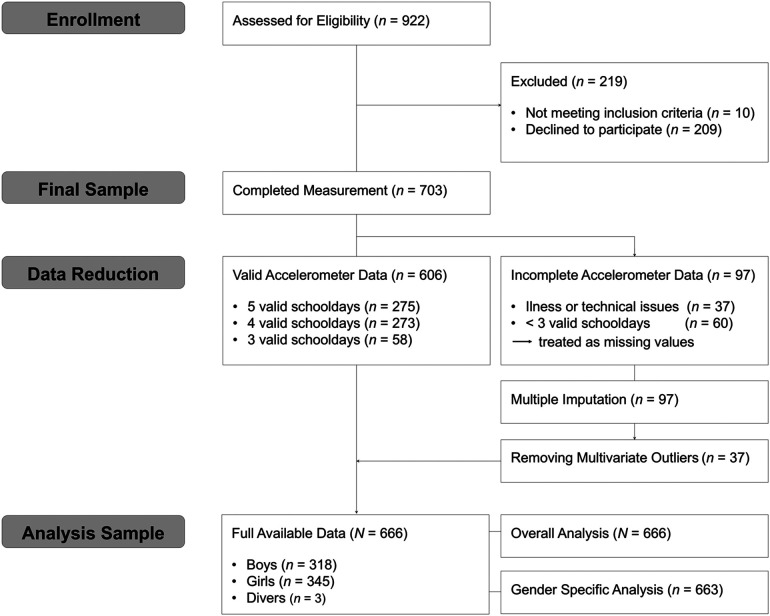
Participant flowchart from enrollment to final analysis sample (adapted from CONSORT guidelines). *Note:* A valid school day was defined as ≥10 hours of wear time (excluding sleep).

A total of 922 students from 45 school classes were invited to participate in the study (see Figure 1). Only 7th-grade students were invited, as the Active School project—on which the present study is based—is a longitudinal study designed to initiate data collection in grade 7 in order to enable follow-up across secondary school and minimize dropout. Of these, 703 students agreed to participate, while 209 declined due to motivational or organizational reasons. Additionally, 10 students were excluded because they were unable to participate in regular physical activity due to injury, which constituted an exclusion criterion. From the original dataset (*N* = 703), 37 adolescents (5.3%) had missing accelerometer data, primarily due to illness or technical issues during measurement. In addition, 60 adolescents (8.5%) had invalid accelerometer data, and these data were therefore treated as missing (53). The dataset was then screened for univariate outliers to identify and remove potential measurement errors. As Little's missing completely at random (MCAR) test (54) was significant, *χ*²(324) = 633.15, *p* < .001, indicating that the data were not MCAR, multiple imputation was employed to handle the remaining missing values (55). In line with recommendations (56), the imputed dataset was examined for multivariate outliers, identified using the Mahalanobis distance, with a critical threshold set at *χ*²(30) = 59.70, *p* < .001. Based on this, 37 cases were excluded for exceeding the threshold. The final analysis sample consisted of 666 7th-grade students (*M* = 13.27 years, *SD* = 0.55, range = 11-15 years), including 51.8% girls (*n* = 345), 47.7% boys (*n* = 318), and 0.5% (*n* = 3) who identified as diverse. All students were included in the overall analysis. For gender-specific analyses, only data from students who identified as girls or boys (*n* = 663) were included, as the number of students who identified as diverse (*n* = 3) was too small to allow meaningful statistical comparisons.

### 2.3 Measures

#### 2.3.1 Adolescents characteristics

To accurately describe the sample, a range of anthropometric variables (e.g., height, weight, age, gender) and demographic factors (family health climate and socioeconomic status) were recorded. Height was measured to the nearest 1 cm using a portable stadiometer (Anthrofex, NutriActiva, Germany), and weight to the nearest 0.1 kg with a digital scale (EBS002K, Esperanza). Body mass index (BMI) was then calculated based on participants’ age and gender (57). The remaining variables were assessed via questionnaire. The Family Health Climate Scale (FHC) (58) was used to evaluate family health climate, and the Family Affluence Scale III (FAS III) (59) to determine socioeconomic status. The FHC Scale has been validated in a German setting, demonstrating good reliability and construct validity (58). The FAS III has likewise shown good validity (59), including significant correlations with parent-reported income (60), and has previously been applied in samples of Swiss adolescents (61–63).

#### 2.3.2 Physical activity

Students’ physical activity intensities were measured using GENEActiv accelerometers (ActivInsights Ltd., Kimbolton, Cambridgeshire, UK), a lightweight, waterproof device worn on the non-dominant wrist. These accelerometers provide valid estimates of PA intensities among adolescents (64). Additionally, wrist-worn accelerometry is preferred by adolescents, increasing compliance (65, 66). Recording started and ended at midnight, and accelerometers were initialized with a sampling frequency of 87.5 Hz. Data was processed into 1-second epochs using the R-package GGIR (67). To distinguish sedentary time from sleep, GGIR's nocturnal sleep detection algorithm was applied. Students were instructed to wear accelerometers 24 h/day for nine consecutive days, with the first and last measurement days excluded due to reactivity concerns (68). However, for the present analyses, only school day data were considered, as these were the primary focus of the study. Analyses were therefore based on up to five complete school days (Monday to Friday). Accelerometer data were considered valid if participants accumulated at least 10 hours of wear time per day (excluding sleep), and daily PA averages were computed for participants with at least three valid schooldays, based exclusively on valid measurement days (69). For participants with fewer than three valid schooldays, average values were set to missing in line with methodological recommendations and subsequently imputed to preserve sample size and minimize bias related to complete-case analyses (53). Proposed cut-points for the non-dominant wrist (64) were used to estimate time at different activity intensities: IN, LPA, and MVPA. PA intensity data were analyzed for specific segments: PE, recess, classroom time, entire school time, and leisure time. For this segmentation, accelerometer data were aligned with each participant's individual school schedule. Recess was defined as scheduled mid-morning or afternoon breaks of ∼15–30 min; short transitions (e.g., 5-minute breaks between lessons) were not counted as recess time. Entire school time covered the period from start to end of the school day, excluding the lunch break, and including PE, recess, and classroom time. Leisure time comprised all waking hours before and after school on weekdays, excluding sleep. To enable comparability across participants despite differences in accelerometer wear time and segment duration, PA outcomes were calculated as percentages of segment time (40).

### 2.4 Procedure

Measurements were carried out at two time points. At t1, adolescents were personally provided with GENEActiv accelerometers in the classroom. Trained research assistants explained how to handle the devices. To motivate daily use and discourage removal, adolescents were informed about participation incentives (e.g., bowling voucher or trampoline park admission) for consistent wear. Simultaneously, body height and weight were measured. At t2, nine days later, accelerometers were collected in the classroom. Subsequently, adolescents completed a paper-and-pencil questionnaire including demographic variables. Two research assistants were present to answer questions.

### 2.5 Statistical analysis

To examine potential differences in adolescents’ MVPA levels between school time and leisure time, a paired-samples *t*-test compared MVPA levels across the entire school time and leisure time (aim 1). To ensure comparability across segments of varying duration, PA intensities were normalized and expressed as the percentage of time spent per segment (40).

To analyze adolescents’ PA behavior across school segments (PE, recess, classroom time) and evaluate compliance with school-based PA recommendations (aim 2), activity levels (IN, LPA, MVPA) were analyzed descriptively. Compliance with PA recommendations (i.e., on average ≥50% MVPA during PE, ≥40% during recess, and ≥30 min across entire school time) was examined by calculating the number of participants meeting each criterion. In addition to percentages, MVPA was expressed in absolute minutes to assess compliance with the 30-minute recommendation. For a more detailed comparison between segments, differences in MVPA across school segments were analyzed using one-way repeated-measures ANOVA with Bonferroni-corrected *post hoc* tests.

To examine gender differences in PA behavior across segments (aim 3), all analyses were also conducted separately for boys and girls. Independent-samples *t*-tests assessed gender differences in PA behavior across the segments; chi-square tests examined gender differences in the proportion meeting recommendations. All calculations, including outlier analysis, were performed using SPSS version 28 (IBM Corp., 2021). The level of significance was set at *p* < .05. Cohen's *d* and Cramer's *V* were reported using standard thresholds for small, medium, and large effects (Cohen's *d* = 0.20, 0.50, 0.80; Cramer's *V* = .10, .30, .50) (70).

## 3 Results

In a first step, sample characteristics were examined in more detail, as shown in Table 1. BMI values were within the expected range for this age group, with girls (*M* = 20.58, *SD* = 3.57) showing significantly higher values than boys (*M* = 19.91, *SD* = 3.34). On average, participants reported medium to high socioeconomic status. Interestingly, girls reported a significantly lower family health climate compared to boys. In addition, no significant differences were observed between compliant (≥ 3 valid accelerometer days) and non-compliant participants with respect to sex, age, or BMI (all *p*s > .05).

**Table 1:** Descriptive statistics and gender differences in sample characteristics by gender (M, SD, t, p, d).

Sample characteristics	Overall	Boys	Girls	Gender differences		
	(*N* = 666)	(*n* = 318)	(*n* = 345)	*t*(661)	*p*	*d*
Age (years)	13.27 (0.55)	13.28 (0.53)	13.26 (0.56)	0.18	.855	0.04
Height (cm)	163 (7.85)	165 (8.94)	161 (6.30)	5.46	<.001	0.52
Weight (kg)	54.15 (11.51)	54.46 (12.14)	53.88 (10.94)	0.64	.522	0.13
BMI (kg · m^−2^)	20.27 (3.47)	19.91 (3.34)	20.58 (3.57)	−2.47	.014	−0.22
Socioeconomic status (0–13)	9.71 (1.82)	9.77 (1.90)	9.66 (1.72)	0.75	.455	0.06
Family health climate (0–3)	1.93 (0.52)	1.97 (0.52)	1.89 (0.52)	2.01	.046	0.15

*d* = Cohen's *d* (positive values indicate higher scores for boys, negative values indicate higher scores for girls).

**Figure 2:**
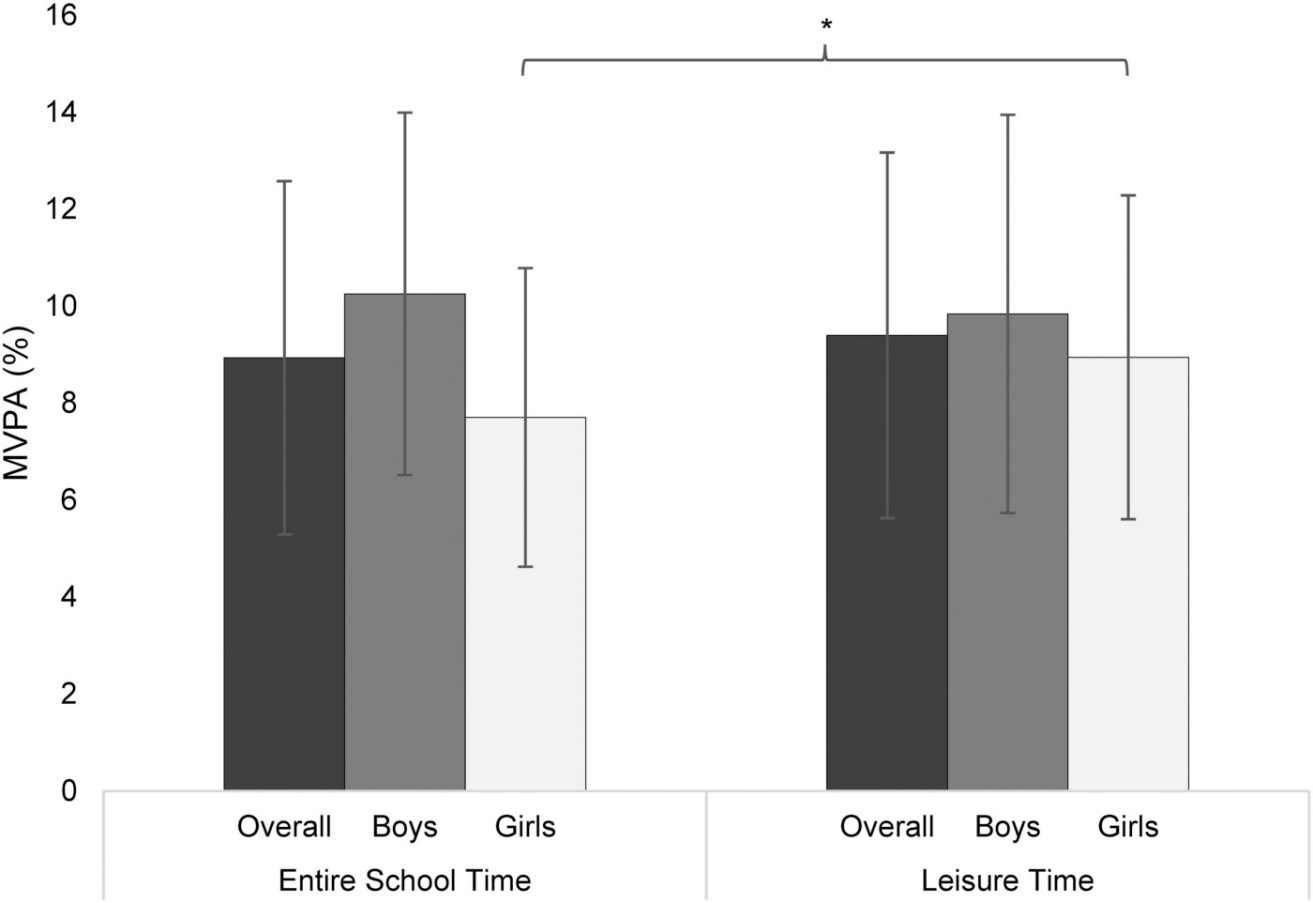
MVPA (%) during school and leisure time by gender. *Note:* Estimated means and error bars (representing the standard deviation) of adolescents’ MVPA (%) during entire school time and leisure time, shown separately for the overall sample, boys, and girls. * *p* < .05.

Overall, MVPA levels were comparable between leisure (*M* = 9.40, *SD* = 3.77) and school time (*M* = 8.94, *SD* = 3.64), with no significant differences for the total sample, *t*(665) = 0.78, *p* = .467, *d* = 0.11. When analyzed by gender, results also revealed no significant difference for boys, *t*(317) = −0.51, *p* = .633, *d* = −0.06. However, girls accumulated significantly more MVPA during leisure time than during school time, *t*(344) = 2.89, *p* = .001, *d* = 0.33, suggesting they were more active outside the school setting (see Figure 2). This pattern is supported by the analysis of leisure-time PA, which showed largely comparable activity profiles between girls and boys. Specifically, no significant gender difference was found in IN during leisure time, *t*(661) = 1.98, *p* = .101, *d* = 0.15. While girls engaged in more LPA than boys, *t*(661) = −5.88, *p* = .001, *d* = −0.45, boys accumulated slightly more MVPA, *t*(661) = 3.09, *p* = .009, *d* = 0.24.

Zooming in on school time segments (see Table 2), a clear and consistent pattern emerged across all segments, with IN accounting for the largest proportion of time. The percentage of time spent inactive ranged from its lowest during PE (39.50%) to its highest during classroom time (75.83%). In contrast, MVPA accounted for the smallest proportion of time in all segments, except for PE, where LPA (29.91%) and MVPA (30.59%) were more evenly balanced.

**Table 2:** Descriptive and inferential statistics of PA by segment: % time (M, SD), gender differences, and compliance (n, %) with school PA recommendations.

PA behavior (%, *SD*)	Overall	Boys	Girls	Gender differences		
	(*N* = 666)	(*n* = 318)	(*n* = 345)	*t*(661)	*p*	*d*
Recess						
IN	46.60 (15.34)	41.45 (15.73)	51.37 (13.31)	−8.79	<.001	−0.68
LPA	34.60 (10.56)	36.60 (11.13)	32.79 (9.67)	4.73	<.001	0.37
MVPA	18.80 (10.00)	21.95 (11.32)	15.84 (7.48)	8.25	<.001	0.64
Physical education						
IN	39.50 (16.22)	35.56 (16.88)	43.21 (14.69)	−6.27	<.001	−0.48
LPA	29.91 (8.36)	30.56 (8.96)	29.27 (7.72)	2.01	.291	0.13
MVPA	30.59 (12.38)	33.88 (13.26)	27.52 (10.63)	6.87	<.001	0.53
Classroom time						
IN	75.83 (6.79)	73.52 (6.86)	77.96 (6.00)	−8.91	<.001	−0.69
LPA	18.48 (4.78)	20.01 (4.87)	17.08 (4.24)	8.27	<.001	0.64
MVPA	5.69 (3.02)	6.47 (3.32)	4.96 (2.50)	6.64	.003	0.52
Entire school time						
IN	70.49 (7.76)	67.69 (7.76)	73.11 (6.78)	−9.63	<.001	−0.75
LPA	20.57 (4.69)	22.06 (4.75)	19.18 (4.20)	8.26	<.001	0.64
MVPA	8.94 (3.64)	10.25 (3.73)	7.71 (3.07)	9.62	<.001	0.75
Leisure time						
IN	71.35 (7.25)	71.94 (7.68)	70.85 (6.76)	1.98	.101	0.15
LPA	19.25 (4.51)	18.21 (4.49)	20.21 (4.33)	−5.88	<.001	−0.45
MVPA	9.40 (3.77)	9.85 (4.11)	8.94 (3.34)	3.09	.009	0.24
Compliance (*n*, %)	Overall	Boys	Girls	Gender Differences		
	(*N* = 666)	(*n* = 318)	(*n* = 345)	*χ²*(1)	*p*	*V*
Meeting PA recommendations						
Recess (40% MVPA)	29 (4.3)	24 (7.5)	5 (1.4)	15.43	<.001	0.15
PE (50% MVPA)	37 (5.5)	33 (10.3)	4 (1.1)	28.62	<.001	0.21
Entire school time (30 min MVPA)	330 (49.5)	206 (64.5)	124 (35.9)	54.62	<.001	0.28

*d* = Cohen's *d* (positive values indicate higher scores for boys, negative values indicate higher scores for girls).

**Figure 3:**
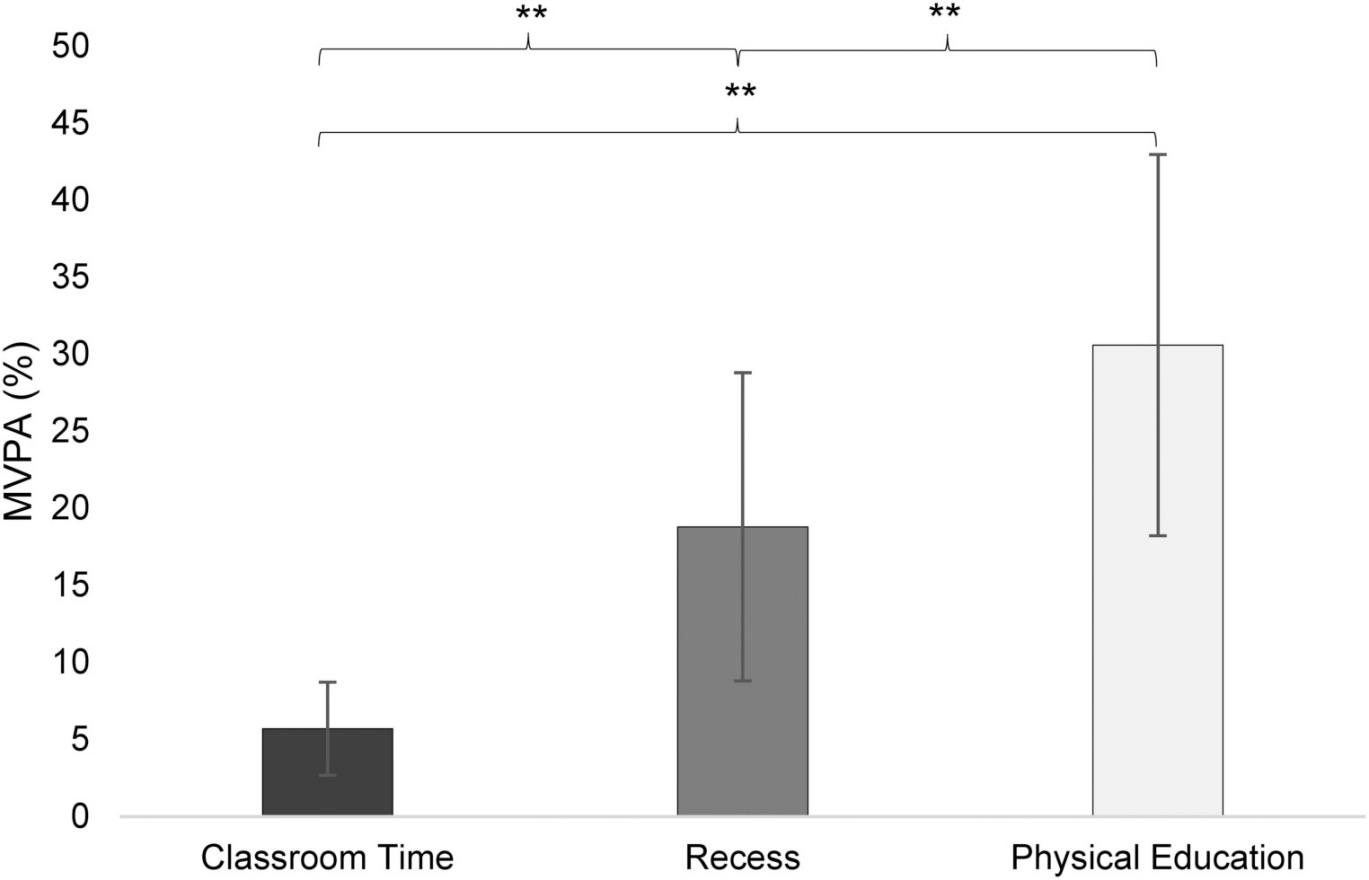
MVPA (%) across school segments in the overall sample. *Note:* Estimated means and error bars (representing the standard deviation) of adolescents’ MVPA (%) during school time segments (classroom time, recess, PE) shown for the overall sample. ** *p* < .001.

When comparing MVPA across school segments (see Figure 3), ANOVA revealed a significant main effect of time segments, *F*(1.79, 1189.55) = 1543.64, *p* < .001, partial *η*² = .696. MVPA followed a consistent gradient, with the highest during PE, intermediate during recess, and the lowest during classroom time (all *post hoc* comparisons, *p* < .001). This pattern was observed for both boys, *F*(1.81, 576.6) = 699.92, *η*² = .684, and girls, *F*(1.62, 557.6) = 953.73, *η*² = .732, with all pairwise differences being significant (*p* < .001). Consistent with this gradient, classroom time showed the lowest MVPA levels and the highest proportion of IN, indicating that sedentary behavior was predominated in this segment.

Across all school segments, pronounced gender differences were evident. Girls spent significantly more time inactive and less time in both LPA and MVPA compared to boys in recess, classroom time, and entire school time (*p* < .001). During PE, girls were more inactive and engaged in less MVPA than boys, *t*(661) = −6.27 and 6.87, *p* < .001, *d* = −0.48 and 0.53, respectively. However, no significant differences were found for LPA, *t*(661) = 2.01, *p* = .290, *d* = 0.13.

Analysis of compliance with school-based PA recommendations (see Table 2) highlighted substantial shortfalls across all segments. The average proportion of MVPA during PE (30.59%) was well below the recommended 50%, with only 37 adolescents (5.5%) meeting this benchmark. Similarly, average MVPA level during recess was 18.8%, below the 40% recommendation, and only 29 participants (4.3%) achieved this target. In contrast, compliance improved when considering the entire school day, with nearly half of adolescents (330; 49.5%) meeting the guideline of at least 30 min of MVPA. Results further indicate that girls were generally less likely than boys to meet school-based PA recommendations. Only a very small number of girls fulfilled the targets for PE and recess, and while 35.9% met the recommendation for the school day, this remained substantially lower than the 64.5% among boys. These gender disparities were statistically significant across all three recommendations (*χ*²-tests, *p* < .001).

## 4 Discussion

This study aimed to examine PA behavior among Swiss secondary school students across different time segments. Specifically, we (1) compared PA levels during leisure time and school time, (2) assessed PA patterns across school segments and compliance with school-based PA recommendations, and (3) examined gender differences.

Contrary to our first hypothesis, no significant difference was found between PA levels during entire school time and leisure time. Regarding the second hypothesis, analysis showed that neither PE nor recess provided sufficient MVPA for most students to meet recommended levels, and only about half of the adolescents reached the overall school-based recommendation of at least 30 min of MVPA per day. In line with our third hypothesis, gender differences were evident across all school segments: girls accumulated significantly less MVPA and more IN than boys.

### 4.1 PA during leisure time vs. entire school time

The hypothesis that adolescents would engage in more MVPA during leisure time than school hours was not confirmed. This contrasts with previous international research suggesting that adolescents are generally more active during leisure time (13–15). In the present sample, MVPA levels were comparable across both settings, indicating that the school context does not inherently limit PA opportunities. However, in both settings, IN accounted for the largest proportion of time, underscoring the need to increase overall activity intensity among the study population. Gender-specific analyses revealed that boys showed similar MVPA levels in both settings, while girls were significantly more active during leisure time. This finding aligns with prior research showing that girls tend to “compensate” for lower PA at school by being more active in their free time (71). This may suggest that the current school environment in Switzerland is more aligned with boys’ movement preferences. This is consistent with studies showing that, especially during recess and PE, activities often cater more to boys, for instance, through the dominance of team ball games and the facilities provided (49, 50, 72, 73). Such settings may limit girls’ active participation, as they tend to prefer socially supported, cooperative, and structured activity formats (74–76) that are embedded within existing educational routines. These findings underscore the need to reflect on how Swiss schools can better accommodate diverse movement needs, especially those of girls.

### 4.2 PA patterns across school segments and recommendation compliance

In all school segments, IN accounted for the largest share of time, while MVPA made up the smallest, except for PE. This aligns with international studies showing that school settings are often marked by low PA and high sedentary behavior (14, 29, 34, 39, 40, 71). Despite high levels of IN, 49.5% of the study participants still meet the recommended 30 min of MVPA during the school day. This proportion is higher than in comparable international studies (40) and exceeds the findings of a Swiss study on primary school children, in which only 14% met the same recommendation (22). Notably, this result is surprising, as PA typically declines with age (77). However, this finding should be interpreted with caution. On the one hand, the Swiss study, conducted at the primary school level, used hip-worn ActiGraph devices, whereas the present study relied on wrist-worn GENEActiv monitors, which typically yield lower estimates of inactivity and higher estimates of MVPA (78). On the other hand, the Active School project sample was not randomly selected and may have attracted schools with above-average PA engagement. Comparisons must also be made cautiously, as current recommendations are based on absolute MVPA minutes. Differences in school schedules (e.g., morning-only instruction, fewer weekly hours in primary school) may affect potential MVPA accumulation (79). To improve comparability, it has been suggested to express PA recommendations relative to time, such as minutes per hour or percentage (40). While approximately half of the study participants meet school-time PA recommendations, the rest remain insufficiently active. Segment-specific insights enable targeted PA promotion throughout the school day.

Classroom time showed significantly lower MVPA levels compared to recess and PE. This is unsurprising, as classrooms are designed for cognitive learning, not PA. Swiss data show that 75.83% of classroom time is spent inactive, with only 5.69% devoted to MVPA, a pattern consistent with international findings (39). As the largest segment of the school day, classroom time is a key setting for potential PA accumulation (80). Given the remarkably low MVPA levels in the present Swiss sample, there is clear room for improvement. Integrating PA into lessons has been shown to boost activity (81). For example, adolescents engaging in activity breaks are up to 75% more likely to meet the 30 min MVPA recommendation (82). This is particularly important given growing evidence that both regular physical active learning and specifically designed active breaks (83) not only increase physical activity levels (46) but also enhance cognitive function (84), learning outcomes (85), and academic achievement (86). To address this untapped potential, policies that provide daily PA opportunities during classroom time may be particularly valuable in the Swiss school context (31), as evidence suggests that PA policies targeting instructional time on a regular basis are especially effective in increasing MVPA (87, 88). Such policies should further prioritize short, high-intensity activity breaks, rather than solely increasing activity volume through low intensive physically active learning or standing desks (89), supported by ready-to-use materials for teachers (90) and classroom-based reminder systems.

During recess, study participants engaged in MVPA for 18.80% of the time, a proportion broadly consistent with international findings (39), yet still below the 40% recommendation, indicating substantial room for improvement. International evidence suggests that recess-focused policies, such as extending recess duration (91), maximizing access to PA spaces (92), and offering a variety of activity options (31), can effectively increase PA. In the Swiss context, where recess typically lasts 15–30 min, optimizing school timetables to enable longer breaks (e.g., 30 min) in the morning and afternoon, alongside consistent and unrestricted access to sports facilities (e.g., outdoor fields or gyms), may be particularly beneficial. In addition, providing structured activity options during recess, potentially supported by playground supervisors, could further promote engagement. Finally, low-cost strategies, such as ensuring access to play equipment (91) or mobile phone bans (93), may help reduce sedentary behavior and increase PA during recess.

PE showed the highest MVPA proportion among all school segments (30.59%) but remained below the international average of 34.7% (37). Accordingly, only 5.5% of the study participants met the 50% MVPA guideline. Potential explanatory factors include suboptimal lesson structure (43), low motivation, and students’ perceived competence (94). Moreover, not all PE activities promote high-intensity movement-fitness training equally, and team games typically result in more MVPA than dance or technical instruction (41). This highlights the need to monitor lesson content and optimize PE through effective class management, support for less skilled students, and active task design (95). Such optimization requires sustained effort from PE teachers to continuously improve PE programs and may be supported at the policy level by annual evaluation of PE programs, which international research suggests can increase PA during lessons (96).

Overall, the results indicate that substantial opportunities for PA are lost across all school segments in the Swiss context, underscoring the need for a balanced, comprehensive school approach to PA promotion through targeted policies. However, translating such policies into practice is challenging (31), as successful implementation requires the coordinated involvement of supportive school principals and teachers, who play a central role in implementation (97). To promote sustainable adoption, an effective and transferable strategy may be to engage influential teachers as PA champions, facilitating policy implementation in collaboration with school principals and the wider teaching community (97). Furthermore, baseline needs assessments are valuable for enabling schools to identify context-appropriate policies, as PA promotion is not a one-size-fits-all approach (28, 31). From a resource perspective, a phased implementation of policies, starting with one school segment and gradually expanding to others, combined with adequate financial support, may further enhance feasibility and sustainability (98).

### 4.3 Gender differences in PA behavior

The present study shows that girls were significantly less likely than boys to meet the school-based PA recommendation (35.9% vs. 64.5%). In PE and recess specifically, only 1.1% and 1.4% of girls met the segment-specific MVPA recommendations. This disparity may be influenced by factors such as social norms, perceived competence, and the types of activities offered (99). In PE and recess, girls may also feel inhibited or disengaged due to competitive dynamics with boys, as they often report lower self-efficacy in competitive activities (100). To counter this, schools should offer PE and recess activities that are diverse and responsive to girls’ preferences (72). Self-selected, non-competitive, and socially supportive options—such as dance-based activities, fitness or movement circuits, yoga, walking or running groups, and cooperative games without performance-based evaluation—may be especially effective (101, 102), as they promote autonomy, enjoyment, and social relatedness rather than competition and comparison (76). Another possibility would be to offer PE activities in gender-segregated settings, as studies suggest that girls report higher perceived competence, greater enjoyment, and increased activity levels in single-gender classes compared to coeducational ones (103). In the Swiss context, gender-segregated PE lessons are only partly offered and depend on school-specific organization, but may nevertheless represent a feasible strategy in certain settings. However, it is also important to recognize that 35.5% of boys did not meet the school-based PA recommendation. This also highlights the need to move beyond binary assumptions and consider individual variability within gender groups (104), acknowledging the existence of both less active boys and girls. This underscores the importance of identifying gender-specific at-risk subgroups (105) and characterizing them through analyses of intra-individual factors influencing MVPA across gender, while considering their context-specific relevance (106). Research suggests that adolescents with lower psychological or physiological indicators tend to show reduced MVPA, regardless of gender (41, 73). Future studies should therefore conduct more fine-grained analyses to better understand the determinants of low PA engagement within and across gender groups. Nevertheless, from a practical perspective, this underscores the importance of student-based needs assessments in the development of PA promotion strategies, as evidence shows that interventions tailored to the specific needs of less active groups—particularly girls—can lead to more sustainable increases in PA (107).

### 4.4 Strengths and limitations

One key strength of this study is the objective assessment of adolescents’ PA using GENEActiv accelerometers, which provides valid, reliable data and reduces biases associated with self-reports (108). Additionally, the alignment of activity data with individual timetables allowed for precise segmentation into PE, recess, and classroom time (109). Despite these strengths, the study also presents some limitations that should be considered when interpreting the findings and when planning future research.

Firstly, despite incentives, approximately 20% of eligible students chose not to participate, an issue commonly reported in adolescent studies (110), which may have resulted in a selective study sample. Recruiting this age group remains challenging. Future studies should therefore address these recruitment challenges by focusing on clear, timely communication and provide rewards immediately after data collection to boost engagement (111). Closer teacher involvement is also key, as their relationship with students helps convey the study's relevance more credibly (112). Additionally, once adolescents have decided to participate, it is essential to maintain their compliance, for example, through the use of reminder systems (113). Secondly, this study included only 7th-grade students, which limits the generalizability of the findings to older adolescents in higher grades, as PA patterns change substantially across adolescence (7). Thirdly, no detailed contextual data were collected on the specific types of activities during PE and recess, limiting the interpretation of intensity differences. Including such contextual information, alongside assessments of activity across multiple weeks, would enhance the explanatory value of PA data and support more meaningful comparisons (41).

## 5 Conclusion

This study examined PA patterns among Swiss adolescents across different school segments and by gender, providing a fine-grained analysis that addresses a critical evidence gap and informs tailored school-based PA policies and recommendations for key stakeholders. The results show that valuable PA time is lost across all school segments, including classroom time, recess, and PE with segment activity recommendations clearly not being met. Furthermore, girls were consistently less active than boys across all school segments. These findings highlight the importance of a balanced PA promotion strategy across all school segments. Practical implications could include daily intensity-promoting PA breaks during classroom time, optimization of school timetables to ensure at least 30 min recess periods, and regular evaluation of PE programs to increase active lesson time. To support effective and sustainable implementation, a comprehensive school approach is recommended, integrating multiple stakeholders such as supportive principals, engaged teachers, and designated PA champions, who can act as key facilitators and coordinators of PA implementation within the school. As girls were consistently less active than boys, gender-responsive strategies within the segments are particularly needed, such as self-selected, non-competitive, and socially supportive activity options, greater choice, and—where appropriate in physical education—single-gender settings may be especially beneficial. At the same time, less active boys must not be overlooked; addressing the needs of all inactive subgroups is essential. Future research should therefore explore individual-level factors influencing adolescents’ PA participation, including students’ individual interests and preferences, to better inform targeted strategies. Such measures can help schools to move beyond isolated interventions and instead create an inclusive culture of activity throughout the entire school day.

## Data Availability

The raw data supporting the conclusions of this article will be made available by the authors, without undue reservation.
